# Quantification of Accidental Gluten Contamination in the Diet of Children with Treated Celiac Disease

**DOI:** 10.3390/nu13010190

**Published:** 2021-01-09

**Authors:** Chiara Monachesi, Anil K. Verma, Giulia N. Catassi, Tiziana Galeazzi, Elisa Franceschini, Valentina Perticaroli, Elena Lionetti, Carlo Catassi

**Affiliations:** 1 Celiac Disease Research Laboratory, Polytechnic University of Marche, 60123 Ancona, Italy; anilkrvermaa@gmail.com (A.K.V.); t.galeazzi@staff.univpm.it (T.G.); 2 Division of Pediatrics, DISCO Department, Polytechnic University of Marche, 60123 Ancona, Italy; giulia.catassi@gmail.com (G.N.C.); elisa.franceschini3@gmail.com (E.F.); vperticaroli92@gmail.com (V.P.); mariaelenalionetti@gmail.com (E.L.); c.catassi@staff.univpm.it (C.C.); 3 Mucosal Immunology and Biology Research Center, Division of Pediatric Gastroenterology and Nutrition, Massachusetts General Hospital, Boston, MA 02114-2696, USA

**Keywords:** celiac disease, gluten traces, gluten exposure, diet therapy, treatment compliance, R5 ELISA

## Abstract

A strict gluten-free diet is extremely difficult to maintain. Protracted ingestion of gluten traces (>10 mg/day) is sufficient to cause significant damage in the architecture of the small intestinal mucosa in patients on treatment for celiac disease. The aim of this study was to directly measure the level of contaminating gluten in the daily diet of celiac children following a gluten-free diet. From April 2019 to December 2019, celiac disease children (2–18 years old) on a gluten-free diet for ≥6 months were offered to participate in this prospective-observational study. Patients and their caregivers were invited to provide a representative portion (about 10 g) of all meals consumed during a 24-h period. Participants were requested to weigh all ingested food and report items in a 24-h food diary. The gluten content was quantified by the R5 sandwich enzyme-linked immunosorbent assay method. Sixty-nine children completed the protocol. Overall, 12/448 (2.7%) food samples contained detectable amounts of gluten; of them, 11 contained 5–20 ppm and 1 >20 ppm. The 12 contaminated food samples belonged to 5/69 enrolled patients. In these 5 children, the daily gluten intake was well below the safety threshold of 10 mg/day. The present findings suggest that in a country characterized by high celiac disease awareness, the daily unintended exposure to gluten of treated celiac children on regular follow-up is very low; reassuringly, the presence of gluten traces did not lead to exceed the tolerable threshold of 10 mg/day of gluten intake in the gluten-free diet.

## 1. Introduction

Celiac disease (CD) is a chronic immune-mediated enteropathy triggered by the ingestion of gluten in genetically predisposed individuals. A strict and permanent gluten-free diet (GFD) is the only effective treatment for CD. The GFD determines disappearance of celiac-related symptoms and serum autoantibodies, recovery of intestinal mucosa, and prevention of long-term complications [1].

Both children and adults with CD are highly sensitive to the toxic effects of gluten exposure. It has been shown that the protracted ingestion of gluten traces (>10 mg/day) is sufficient to cause significant damage in the architecture of the small intestinal mucosa in patients on treatment for CD. Based on this threshold, a maximum tolerable amount of gluten of <20 parts per million (ppm) in gluten-free food has been calculated [2], a limit that has been endorsed by the major international regulatory agencies, e.g., the Codex Alimentarius, the US Food and Drug Administration (FDA), and the European Food Safety Authority (EFSA) [3,4,5]. Deviation from the GFD is unfortunately easy, due to both voluntary and inadvertent dietary transgressions. Gluten is indeed a pervasive ingredient that may be used as a protein filler in many commercial food (e.g., sausages, soups, soy sauces, etc.) or may contaminate originally gluten-free products (e.g., oats and legumes) during the production chain.

In recent years, several studies from different countries investigated the level of gluten contamination in foodstuff [6,7,8,9,10,11], but only few data are available on the daily intake of contaminating gluten in treated CD patients.

The aim of the present study was to directly measure the level of contaminating gluten in the diet of CD children followed at our Celiac Center.

## 2. Materials and Methods

### 2.1. Study Group

From April 2019 to December 2019, CD children (2–18 years old) on GFD for ≥6 months attending medical follow-up visits at our Celiac Center were offered to participate in the study. The initial diagnosis of CD was performed according to the European Society for Pediatric Gastroenterology, Hepatology and Nutrition (ESPGHAN) guidelines [12]. Patients who had comorbidities requiring additional dietary restrictions, particularly Type 1 Diabetes, inflammatory bowel diseases, or food allergies, were excluded from participation. Written informed consent was obtained from parents of participating children, and additional written assent was obtained from age-eligible children. The study was conducted in accordance with the principles of the Helsinki Declaration as revised in Fortaleza 2013 and was approved by the ethical committee of the Polytechnic University of Marche (ID # 124827).

### 2.2. Study Design

Participants were encouraged to maintain their usual eating pattern during the diet sampling period. The weekday of diet sampling was randomly assigned at enrollment. Patients and their caregivers were invited to provide a representative portion (about 10 g) of all meals consumed during the 24-h period. They were requested to weigh all ingested food using a kitchen scale and to report the amount, the composition, and other details (including ingredients, food type, place, and time of sampling) of each meal/snack on the 24-h food diary. Each subject was provided with sterile plastic bags and cups to collect food portions. Samples from breakfast, lunch, snacks, and dinner were included. Naturally gluten-free, unprocessed food (e.g., water, milk, fruits, and raw vegetables) were not collected. Samples were given a unique laboratory code, and were stored at −20 °C until analysis.

### 2.3. Methods

#### 2.3.1. Determination of Gluten Content in Food Samples by R5 Ridascreen ELISA

All food samples were processed for gluten content determination by the Ridascreen Gliadin sandwich R5 enzyme-linked immunosorbent assay (ELISA) R-7001 (R-Biopharm, Darmstadt, Germany) at our Celiac Disease Research Laboratory, Polytechnic University of Marche, Ancona. During each run of ELISA, the manufacturer’s guidelines were strictly followed. The Ridascreen R5 ELISA was performed as previously described [6].

#### 2.3.2. Gluten Quantification

The gluten content of analyzed food samples was expressed as ppm. The lower limit of quantification was 5 ppm of gluten. All products with a gluten level higher than 20 ppm were re-extracted and analyzed for a second time.

Finally, we estimated the 24-h amount of gluten consumed by participating children using the following formula to convert ppm of gluten into mg of gluten/day for all the meals with measurable gluten contamination: mg/day gluten = ppm gluten in the food portion × food sample weight (g)/1000.

#### 2.3.3. Determination of Serum IgA Anti-Tissue Transglutaminase Antibody

IgA anti-tissue transglutaminase (anti-tTG) antibody assay was performed in all participating children in our Laboratory by fluorescence enzyme immunoassay ≤30 days prior to the start of the study (normal values <7 U/mL) as part of routine follow-up visits.

### 2.4. Statistical Analysis

Data are presented as medians (range) or percentages, as appropriate. The sample size was estimated on the basis of the expected prevalence of gluten exposure. GraphPad Prism software (version 7, GraphPad Software, La Jolla, CA, USA), and Microsoft EXCEL (v.2010; Microsoft Corp Redmond, Washington, DC, USA) were used for the analysis.

## 3. Results

Of the 94 eligible pediatric CD patients, 25 children were excluded because of concomitant diseases (*n* = 5) or declined participation (*n* = 19) or incomplete collection of samples (*n* = 1). Sixty-nine children completed the protocol. Demographic and clinical data of these patients are shown in Table 1.

No intentional gluten exposure was reported during the 24-h period of diet sampling by these patients and their caregivers.

Each patient provided 7 food samples on average. A total of 448 food samples were provided from these 69 subjects. Samples belonged to the following food categories: “Pasta and bakery products” (46%) including pasta, lasagna, rice, pizza, wraps, crackers, breadsticks, sandwiches, and stuffed focaccia; “Sweet snacks” (26%) including biscuits, cakes, nougats, ice-creams, muesli, waffles, cornflakes, and chocolate tarts; “Meat/fish-based products” (20%) including cooked meat/fish, cold cuts, eggs, cheeses, yogurts, and mayonnaise; “Vegetable-based products” (8%) including cooked vegetables, processed fruits, legumes, and vegetable soups. Meals including foods from more than one food group were assigned to a specific category on the basis of the most represented ingredient. In total, 299 samples were collected at home, 76 at relatives’ home, 61 at school, and 12 at restaurants. Of them, 316 were collected on weekdays and 132 during the weekend. The level of gluten contamination in the analyzed samples according to the different settings of consumption is shown in Table 2.

**Figure 1:**
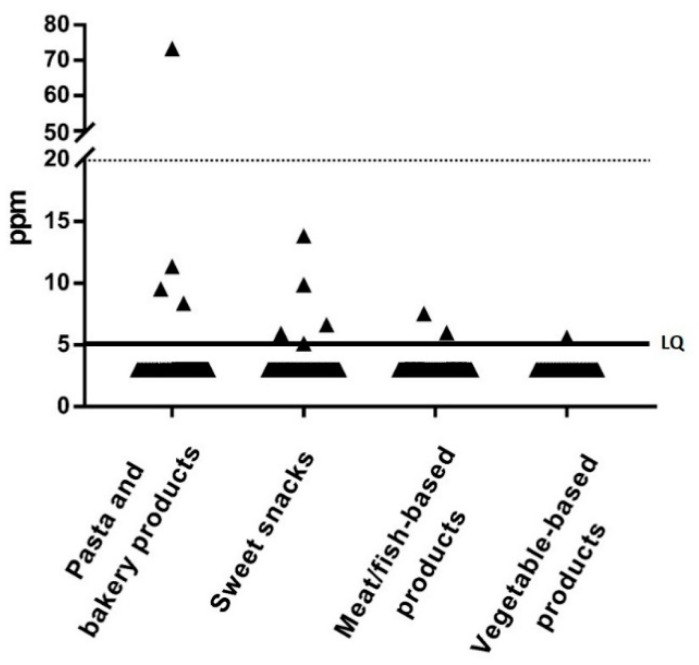
Gluten content in the 448 analyzed samples according to the food group. LQ, limit of quantification (solid line).

Overall, 12/448 (2.7%) food samples showed detectable gluten contamination; of these, 11 contained gluten within tolerable limits (5–20 ppm) and only one contained >20 ppm of gluten (Figure 1).

The median concentration of gluten in positive samples was 8 ppm (range: 5 to 74 ppm). The 12 contaminated food samples were from 5 of the 69 enrolled patients (7%; 1 male and 4 females): 2 patients had only 1 contaminated meal (total level of gluten contamination/day was 1.86 mg and 0.18 mg, respectively), 2 patients had 2 contaminated meals (total level of gluten contamination/day was 0.39 mg and 0.58 mg, respectively), and 1 had 6 contaminated meals (total level of gluten contamination/day was 3.61 mg). Two of these 5 patients showed IgA anti-tTG antibodies positivity: one had 7-fold and the other 1-fold higher levels than the upper normal value (cutoff: 7 U/mL). No significant difference was found in the percentage of anti-tTG antibody positivity according to the presence of gluten contamination in the diet (*p* = 0.664). Three of the 12 contaminated items were from children aged 2–5 years while 9 were from children aged 6–10 years. No contaminated items were found in subjects aged 11–18 years. The only food sample contaminated with more than 20 ppm of gluten was from a 2-year-old female patient, and was prepared and consumed at the grandmother’s home (total level of gluten contamination/day was 1.86 mg). In the 5 children ingesting contaminated foodstuff, the daily gluten intake was always well below the safety threshold of 10 mg/day (3.61, 1.86, 0.58, 0.39, and 0.18 mg/day, respectively).

## 4. Discussion

To our knowledge, this is the first study to quantify the amount of inadvertent gluten exposure in treated CD patients. In our sample of 69 Italian CD children on GFD regularly followed-up, we found that gluten contamination of the GFD was extremely rare (only one food sample showing >20 ppm of gluten out of 448 analyzed) and almost negligible on a quantitative basis. Only 5 out of 69 celiac children (7%) ingested gluten traces during the 24-h test-period, and the total amount of gluten contamination (0.2–4 mg/day) was always well below the tolerable threshold (10 mg/day) in these cases.

A strict GFD is extremely difficult to maintain since gluten may contaminate many different commercial food items. The only method to quantify traces of gluten in the GFD is the analysis of ingested food by a reference analytical method associated with quantification of food portions consumed during a given period of time, e.g., 24 h, as performed in the present study. The ELISA R5 used here is currently classified as a Codex type I method for gluten determination in foods and, therefore, represents the most widely used assay [13]. The R5 antibody accurately detects prolamins of wheat (gliadins), rye (secalins), and barley (hordeins), in both raw flours and processed food products [14]. Limitations of this method are the potential interference of different food matrices with antibody binding, and the poor reliability in measuring hydrolyzed gluten in beer, a dietary component that was not consumed by our pediatric patients. R5 ELISA is the only certified method that has been endorsed by several international agencies including the Codex Alimentarius, US FDA, and the European EFSA [3,4,5].

Our favorable results may be explained by several factors: (a) inclusion of highly compliant patients who are regularly seen at the Celiac Clinic; (b) generalized conformity of GF products marketed in Italy with the international regulations for labeled gluten-free food [6]; (c) high level of awareness of the requirement of the GFD by the general population in Italy, particularly due to the national Celiac Protection law (n.123/2005) and the pro-active role of the Italian Celiac Association that strongly helps families in managing the daily needs of the GFD, for example, by a capillary surveillance of restaurants and pizzerias.

A higher frequency of food samples contaminated with >20 ppm of gluten (3%) was recently reported by Silvester et al. in Canadian adults with CD, however the overall daily intake of contaminating gluten was not reported in that study [15]. Higher rates of poor adherence to the GFD have been reported in studies based on indirect evaluation of contaminating gluten. Stefanolo et al. [16] investigated the patterns of gluten exposure during a 4-week period, as assessed by GIP excretion in urine and stool. These authors reported a high rate of inadvertent gluten exposure in CD patients, with 89% of patients excreting GIP in either stool and/or urine at least once during the four-week period. It should however be noted that the relationship between urinary GIP positivity and the amount of ingested gluten is still unclear. Furthermore, in that study, samples were collected only during the weekend, when people frequently dine out. Syage et al. [17] estimated that the mean daily gluten consumption of children following a GFD was 387 mg/day. These estimates were based on two assumptions, the conversion factor from GIP to gluten ingestion and the equation describing the relationship between the dose of ingested gluten and the morphometric change of the small intestinal mucosa, that have not been verified so far.

A significant proportion of our patients showed positivity of IgA class anti-tTG antibodies determination, a finding that might suggest persisting active disease caused by ongoing gluten ingestion. However, this result does not conflict with the excellent adherence to the GFD that we observed in our study-group. Previous studies in treated celiacs have consistently shown that the correlation between CD serology results and dietary evaluation of compliance to the GFD is poor [18]. Even more importantly, most of our patients (85%) with anti-tTG positivity were investigated during the first two years of GFD treatment. It is well established that normalization of IgA anti-tTG levels after starting the GFD may take longer than two years in a significant proportion of cases [19].

## 5. Strengths and Limitations

The strengths of our study are the direct determination of contaminating gluten, the prospective registration of ingested food, the large sample of analyzed food, and the use of a reference method to quantify gluten in different food matrices. The limitations are the selection bias introduced by investigating children and families highly compliant with the CD follow-up schedule, and the possible modification of the usual dietary behavior in response to the awareness of being under investigation (so called Hawthorne effect). This is an unavoidable bias in dietary prospective studies like ours.

## 6. Conclusions

In a group of Italian children strictly following the CD follow-up program, the daily unintended exposure to gluten was very low, and did not lead to exceed the tolerable threshold of 10 mg/day of gluten intake in the GFD.

## Figures and Tables

**Table 1:** Demographic and clinical data of the study-group.

Variable	CD Children (*n* = 69)
Gender	27 M (39%)/42 F (61%)
Median age (years)	9 (range: 2–18)
Median disease duration (years)	2 (range: 0.5–9)
■ <1 year	27/69 (39%)
■ 1–2 years	13/69 (19%)
■ >2 years	29/69 (42%)
Positive IgA anti-tTG ^1^	27/69 (39%)
■ <1 year after diagnosis	19/27 (70%)
■ 1–2 years after diagnosis	4/27 (15%)
■ >2 years after diagnosis	4/27 (15%)

^1^ IgA anti-tTG, IgA anti-tissue transglutaminase.

**Table 2:** Gluten contamination level according to food samples features.

	Gluten Contamination		
	<5 ppm *n* = 436 (97%)	5–20 ppm *n* = 11 (2.8%)	>20 ppm *n* = 1 (0.2%)
Place of sampling			
■ Home	336 (97%)	9 (3%)	0 (0%)
■ Relatives	73 (96%)	2 (3%)	1 (1%)
■ School	15 (100%)	0 (0%)	0 (0%)
■ Restaurant	12 (100%)	0 (0%)	0 (0%)
Time of sampling			
■ Weekdays	308 (97%)	7 (2%)	1 (0.3%)
■ Weekend	128 (97%)	4 (3%)	0 (0%)

“Home” included all the meals prepared and consumed at home. “Relatives” included meals prepared and consumed at grandparents’ and uncles’ home. “School” included meals prepared at the school canteen. “Restaurant” included meals consumed in restaurants, pizzerias, sandwich shops, and ice-cream parlors.

## Data Availability

The data presented in this study are available on request from the corresponding author.
